# Mechanical Properties Comparison of Isotropic vs. Anisotropic Hybrid Magnetorheological Elastomer-Fluid

**DOI:** 10.3390/polym16091215

**Published:** 2024-04-26

**Authors:** Hammam M. Ananzeh, Rahizar Ramli, Sabariah Julai, Asan G. A. Muthalif

**Affiliations:** 1 Department of Mechanical Engineering, Faculty of Engineering, University of Malaya, Kuala Lumpur 50603, Malaysia; 22070919@siswa.um.edu.my (H.M.A.); sabsz@um.edu.my (S.J.); 2 Department of Mechanical and Industrial Engineering, College of Engineering, Qatar University, Doha P.O. Box 2713, Qatar

**Keywords:** magnetorheological elastomers, magnetorheological fluids, isotropic, anisotropic, hybrid MRE-F, frequency dependence, SMR effect, dynamic properties

## Abstract

Magnetorheological (MR) materials are smart materials that can change their rheological characteristics when exposed to a magnetic field. Such rheological properties include viscosity and dynamic modulus. MR materials have emerged as one of the most efficient smart materials that can modify mechanical and viscoelastic characteristics. Depending on the medium used, MR materials can be classified into two types: magnetorheological fluids (MRFs) and magnetorheological elastomers (MREs). MREs are classified as isotropic or anisotropic based on CIP distribution inside the elastomer matrix. A unique hybrid material incorporating MRE and MRF is constructed in this work to investigate, compare, and the dynamic properties of isotropic, anisotropic, hybrid isotropic, and hybrid anisotropic MREs under various magnetic fields (0, 104, and 160.2 mT). The created samples are subjected to extensive testing, including static and dynamic evaluations. In the static tests, experiments use a compression linear displacement mode with a fixed maximum gap change of 3 mm. The temperature is maintained at a constant level of 24 °C throughout the 40 s test duration for each test, and the magnetic field is incrementally increased by varying the number of magnets, ranging from 0 to 160.2 mT for dynamic qualities using compression oscillations on a dynamic mechanical analyzer (DMA), including frequency and strain-dependent data. These experiments, carried out using sinusoidal shear movements, include an excitation frequency range of 0.1 Hz to 15 Hz while preserving, with a fixed shear strain of 2%.

## 1. Introduction

Magnetorheological (MR) materials are classified as smart materials capable of altering their rheological properties, such as viscosity and dynamic modulus, when exposed to a magnetic field. Depending on the medium used, MR materials can be categorized into two groups: magnetorheological fluids (MRFs) and magnetorheological elastomers (MREs). The fabrication process of the MRE samples involves three steps: mixing, molding, and curing [1]; in magnetorheological fluids (MRFs), magnetic particles are suspended within a carrier fluid. When subjected to an external magnetic field, the MRFs alter their structure from liquid to semi-solid. The broad flexibility of MRF becomes evident through their widespread usage in various applications, including engine mounts, brakes, and dampers [2,3], due to their quiet operation and fast reaction to the magnetic field applied [4]. However, it is important to highlight the limitations associated with MRFs, including challenges like sealing issues and particle sedimentation. As a result, future advancement in MRF-based engineering applications is quite limited.

The sedimentation challenges encountered in MRFs could be successfully addressed using MREs due to their solid-state composition. Various factors impact the MR effect, such as additives, magnetic particle variants, and magnetic field intensity. The arrangement of magnetic particles within the elastomer matrix can be either anisotropic or isotropic, depending on whether an externally induced magnetic field is present or absent during the curing process. Isotropic MREs are formed when cured without an external magnetic field, whereas anisotropic MREs are created in a magnetic field, resulting in a flat MRE sample aligned perpendicular to it [5]. The magnetic particles in isotropic MREs are equally dispersed in the nonmagnetic matrix and cured in the absence of a magnetic field. On the contrary, the curing process of anisotropic MREs involves the presence of an externally supplied magnetic field, resulting in a specific direction and pattern for the dispersion of magnetic particles [6]. Typical anisotropic MREs have a certain MR effect, which depends on the amount of magnetic field strength during the curing process. The alignment of iron particles is proportional to the magnetic field [7]. Also, the anisotropic MREs have a larger MR impact than isotropic MREs, which also have a slower temporal response [8]. MREs 3D printing is a relatively new additive manufacturing process that facilitates the expansion of MRE manufacture. It is a highly successful solution to the problem of anisotropic MREs requiring a magnetic field [9].

Carbonyl iron particles (CIPs) serve as a magnetically permeable component incorporated into the matrix material. MREs can be fabricated with diverse combinations of soft, hard, and coated magnetic particles. Each CIP volume fraction of every type possesses different rheological and mechanical properties. Numerous properties can be influenced in the MREs, such as yield stress, stiffness, and damping. For instance, the stiffness tends to increase with the amplification of the magnetic field and the rise in the percentage of ferromagnetic particles within the natural rubber [1]. Hence, the inclusion of carbonyl iron particles (CIPs) in the elastomeric matrix plays a crucial role in significantly enhancing the stiffness and hardness of MREs. Consequently, it becomes essential to utilize an optimal volume fraction of CIPs to ensure the adequate magnetization of the MREs. Soft magnetic fillers are characterized by low coercivity and high magnetic permeability, making them easily magnetizable and demagnetizable. In contrast, hard fillers possess the ability to retain magnetism over an extended period, even after the removal of the applied magnetic field. This behavior is attributed to their high coercivity and the necessity for larger magnetic fields to achieve saturation magnetization. When the MRE elastomer is subjected to a magnetic field, the magnetizable particles align themselves, forming chains that create a magnetic network within the elastomer. This network can affect the material’s stiffness in several ways [10,11].

This study investigates the mechanical and rheological properties of isotropic and anisotropic hybrid magnetorheological elastomers-fluid (H-MRE-F). For this reason, the fabrication and assessment of the samples are explored. Employing frequency-dependent and displacement measurements, the investigation aims to determine the storage and loss modulus, subsequently analyzing the dependency of MRE samples’ dynamic characteristics on displacement, frequency, and magnetic field strength. These experiments, conducted using a dynamic mechanical analyzer (DMA) employing sinusoidal shear movements, cover an excitation frequency range of 0.1 Hz to 15 Hz while maintaining a fixed strain amplitude of 2%. The experimental findings serve to characterize the performance of MRE samples by explaining the MR effects observed. Eventually, these results could provide essential insights for optimizing the selection of MRE and MRF types to enhance the efficiency of H-MRE-F-based applications.

The structure of this paper is outlined as follows. Section 2 details the experimental methodology, encompassing the materials employed in MRE fabrication, the setup utilized for dynamic testing, and the measurements conducted. Section 3 offers a thorough analysis and discussion of static and dynamic tests, exploring material stiffness and dependencies of storage and loss modulus on frequency sweep and magnetic field variations.

## 2. Materials and Methods 

### 2.1. Material Preparation

Silicone rubber, CIPs, and silicone oil are the main components used to fabricate the MREs in this study. The silicone rubber Zhermack’s Elite Double 32 Fast (Badia Polesine, Italy), known as the elastomeric matrix, has notable properties, such as high fluidity, long-term dimensional stability, and significant elastic recovery as well as a quick setup period of 10–15 min. Because of the silicone’s quick cure time, the sedimentation of magnetic particles during curing is decreased. Furthermore, the exceptional flowability of this silicone allows for mixing without vacuum conditions, simplifying the MRE synthesizing process. The silicone elastomeric matrix is formed by blending a silicone base and catalyst in a 1:1 ratio. Table 1 lists the parameters of the silicone rubber utilized in this study. The carbonyl iron particles (CIPs) are synthesized through the thermal decomposition of iron pentacarbonyl (FeCO_5_) developed by (BASF, Ludwigshafen, Germany), resulting in high magnetic saturation and permeability due to the inclusion of ferromagnetic materials like iron. These inherent characteristics play a crucial role in developing magnetorheological elastomers (MREs), ensuring a high magnetization level in response to an applied magnetic field. Magnetic fillers, such as CIPs, significantly impact the properties of MREs. Incorporating CIPs into the elastomeric matrix can significantly improve MRE stiffness and hardness. As a result, an appropriate volume percentage of CIPs must be used to achieve adequate magnetization of the MREs. The compatibility between magnetic particles (CIPs) and the elastomeric matrix is a critical challenge to fabricating magnetorheological elastomers (MREs). As a result, researchers are looking for new methods to improve the interaction and integration of magnetic particles (CIPs) with the matrix material (elastomer) [11,12].

The CIPs Grade CS Soft Particle with size ranging between 6.0 and 7.0 (μm) and a density of 7.89 (g/cm^2^) is used in this study. Further, CIPs are added at a 20% volume percentage. These values serve as a bridge between adequate magnetization and passive stiffness; the calculation of the CIP mass for the mixture involves using the following formula [12].

(1) $$m_{CIPs}=\rho_{CIPs}\;\left(VF\%\times V_{T}\right)$$

where $\rho_{CIPs}$ is the density of the CIPs, VF% is the volume fraction, and $V_{T}$ is the total volume of the mixture.

**Figure 1:**
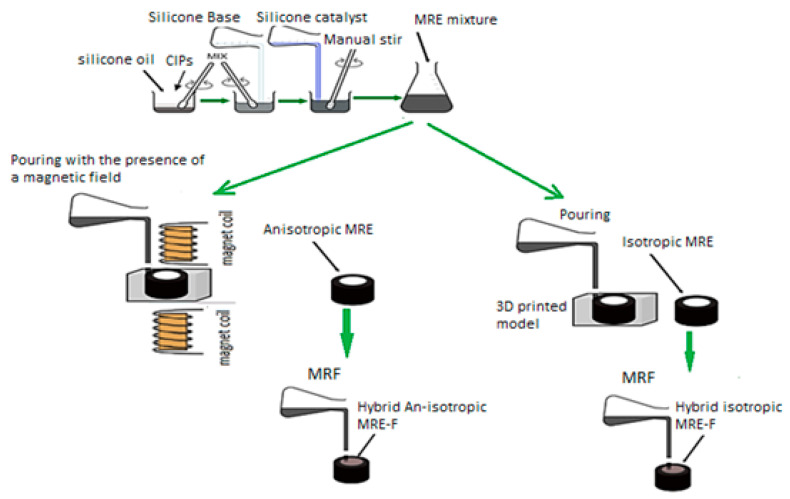
Schematic diagram of the hybrid anisotropic and isotropic fabrication process.

**Figure 2:**
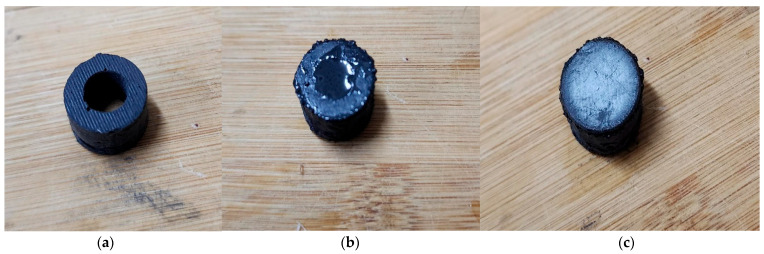
The hybrid sample fabrication, with (**a**) the anisotropic or isotropic MRE, (**b**) the MRE sample after adding the MRF, and (**c**) the final H-MRE-F sample.

Furthermore, a robust MR effect is guaranteed through the formation of a chain-like structure in particle packing, facilitating increased magnetic dipole interaction with nearby particles. Four types of MRE samples were prepared for this study, each was cylindrical shaped with the following dimensions: a diameter of 15 mm, a thickness of 3.5 mm, and a height of 10 mm. These dimensions are taken as per DMA’s guide. Fabrication was executed for isotropic and anisotropic MRE samples, including the hybrid MREs (H-MRE). A detailed depiction of the process is provided in Figure 1, while Figure 2 illustrates the manufacture of H-MRE-F samples. Silicone oil is added with 20% of the total volume in MRE fabrication, which works as a softening agent and decreases the storage order to extract air bubbles [13]. PMX-200 Silicone Fluid 100 cSt obtained from The Dow Chemical Company, United States, was used as a silicone oil component in the experimental materials. The preparation of anisotropic MREs differs from isotropic ones, mainly in the curing process, where a 270 mT magnetic field is applied to the mold’s mixture using coils, The magnetic felid density is measured using a Gauss meter. In the case of the hybrid sample, an MRF was enclosed within the MRE and sealed using 0.8 mm steel on both sides. This formulation comprised CIPs mixed with a 30% volume fraction of silicone oil [12].

### 2.2. Test Setup of Dynamic Mechanical Analyzer

**Figure 3:**
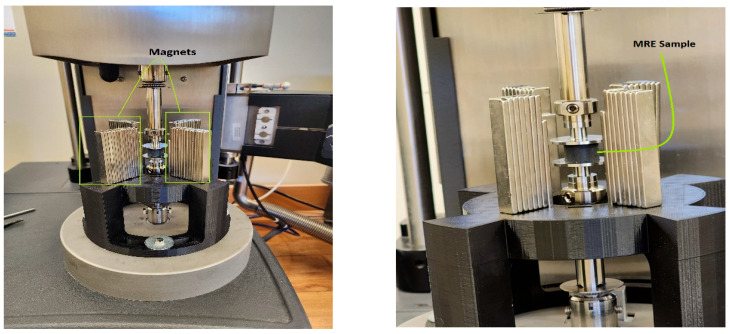
MRE sample position on the dynamic mechanical analyzer.

**Figure 4:**
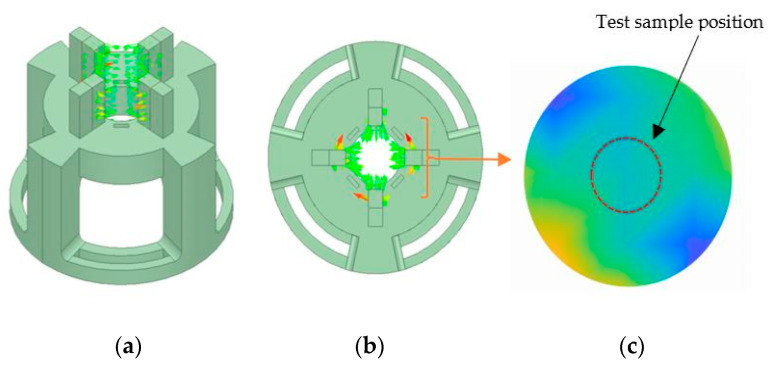
(**a**,**b**) Magnetic vector contour for the magnetic field system by finite element analysis, and (**c**) the distribution of normalized magnetic flux density (measured) and location of the test sample (top view).

Static and dynamic compression tests were conducted using a dynamic mechanical analyzer (DMA) with the model RSA-G2 from (T.A. Instruments, New Castle, DE, USA). A specially designed holder, created through 3D printing, was crafted and attached to the DMA’s base plate. This holder secured the magnets around the MRE specimen during testing. Figure 3 illustrates the experimental setup with an additional adaptor developed to hold magnets. A finite element analysis simulation was performed to investigate the proper selection of magnetic polarity, which directs the maximum flux into the sample, as shown in Figure 4a,b [1], while Figure 4c shows the normalized flux density distribution measured using a Gauss meter F.W. Bell 5180 from (OECO LLC, Milwaukie, OR, USA), and the maximum observed value at total magnetic capacity reached approximately 160.2 mT. The cylindrical samples, with a diameter of 15 mm, height of 10 mm, and thickness of 3.5 mm, were positioned between a lower plate linked to a motor undergoing forced linear oscillations and an immobile upper plate connected to the transducer. The sample dimensions were selected to ensure compatibility with the DMA compression fixture.

For the static test, the experiments were conducted using the DMA in a compression linear displacement mode. The maximum gab change was fixed by 3 mm, the temperature was held constant, and the test duration was 40 s. The magnetic field was increased for each test, starting by increasing the number of magnets from 0 to 160.2 mT.

Moreover, dynamic tests involved a frequency sweep test performed at a constant temperature and strain while varying the frequency. The strain amplitude remained constant at γ = 2%, and the frequency (f) was adjusted within the range of 0.1–15 Hz with increments of 1 Hz. The variations in storage modulus (E’) and loss modulus (E’’) with frequency were assessed at three different magnetic fields, and the results are graphically represented. The objective of the frequency sweep test is to characterize the magnetorheological (MR) effect exhibited by the MR samples. The storage modulus reflects the stiffness of the MR material, illustrating its ability to store deformation energy, whereas the loss modulus indicates the material’s capability to dissipate deformation energy. The process for determining storage and loss moduli from amplitudes and the loss angle is outlined as follows [14]:

(2) $$E^{*}=\frac{\tau_{a}(\omega )}{\gamma_{a}(\omega )},\;\;\;\;E^{\prime }=E^{*}\cos\left(\delta \right),\;\;\;\;E^{\prime \prime }=E^{*}\sin\left(\delta \right)$$

## 3. Results and Discussion

### 3.1. SEM Analysis

**Figure 5:**
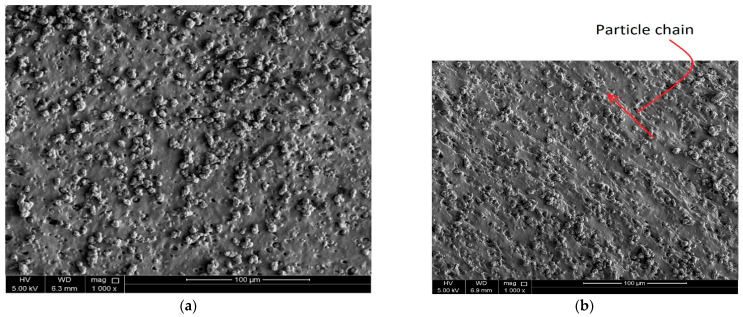
Microstructure images of fabricated MRE samples: (**a**) isotropic sample, (**b**) anisotropic sample (The red arrows indicate the direction of the particle chains).

The microstructures of the isotropic and anisotropic specimens were examined using a (SEM) machine-scanning electron microscope (model Nova Nano SEM 450 and Quanta 200 SEM) with a magnification of 1000 times and an accelerating voltage of 5 kV. As the anisotropic samples’ chain-like features run parallel to the thickness direction, the MRE samples were sliced to a thickness that accommodates the SEM holder. SEM micrographs of MRE samples are shown in Figure 5. Figure 5a depicts the microstructure of isotropic samples, whereas the microstructure of anisotropic samples is depicted in Figure 5b. Notably, the chain structure’s orientation change is attributable to the sample’s placement within the SEM holder during the scanning procedure. The chains in all anisotropic samples align parallel to the sample’s thickness direction. When isotropic and anisotropic MRE samples are compared, the presence of different CIP chains in anisotropic samples is clearly shown.

### 3.2. H-MRE-F and MRE Hysteresis Characteristics

This study focuses on analyzing the effect of external magnets from one corner, where the increase in the number of magnets corresponds to one side of the experimental setup. The magnetic field strength is determined by the quantity of magnets utilized in our study. Specifically, when employing 9 magnets, the magnetic field reaches 129 mT, whereas using 15 magnets results in an increased magnetic flux density of 160 mT.

**Figure 6:**
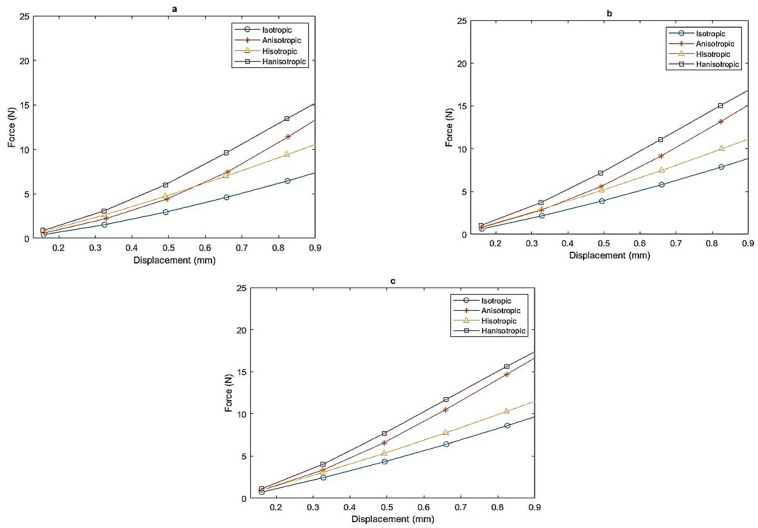
Force–displacement with zero magnets (**a**), nine magnets (**b**), and fifteen magnets (**c**).

Figure 6 illustrates a direct relationship between the number of magnets and the applied force required to achieve the same displacement across all samples. As the number of magnets increases, there is a noticeable increase in force, demonstrating a proportional and predictable effect on the system. This observation underscores the importance of magnet type and arrangement in influencing the force–displacement relationship, providing valuable insights for applications where precise control over magnetic forces is critical.

In addition, a noteworthy distinction emerges when comparing the isotropic, anisotropic, hybrid isotropic, and hybrid anisotropic samples. The graph vividly shows cases of a substantial difference in force required to achieve equivalent displacement between the four. The anisotropic samples exhibit a significantly higher force compared to their isotropic and hybrid isotropic. For instance, to achieve a 0.8 mm displacement, 15.65 N is required for the hybrid anisotropic sample. In contrast, for anisotropic, hybrid isotropic, and isotropic samples, 14.69 N, 10.27 N, and 8.61 N were respectively required to reach the same displacement (as shown in Figure 6c).

Additionally, the hybrid anisotropic sample required the highest force among all samples to achieve the same displacement.

### 3.3. Effective Stiffness

**Figure 7:**
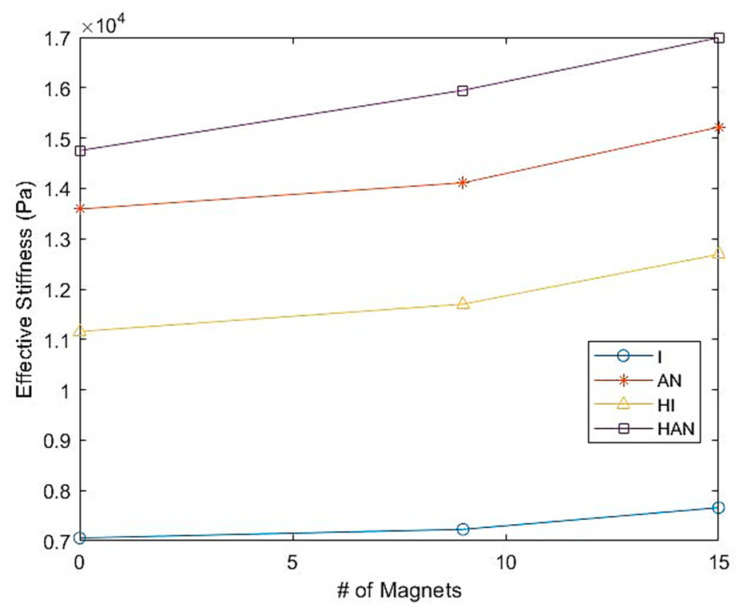
The effective stiffness of H-MRE-F and MRE samples under the influence of a magnetic field.

An effective stiffness study was performed to analyze further the effects of stimulation inputs and magnetic fields on isotropic, anisotropic, and hybrid samples. The following equation can be used to represent effective stiffness:

(3) $$K_{eff=}\frac{F_{dmax}-F_{dmin}}{{∆}_{max}-{∆}_{min}}$$

where *F*_dmax_ and *F*_dmin_ are the forces determined at the ultimate displacement (Δ*max*) and lower displacement (Δ*min*) in a single test, respectively. Figure 7 demonstrates how a magnetic field affects the effective stiffness of MRE samples. As the magnetic field increases, the effective stiffness of all MRE samples increases.

Furthermore, it indicates that anisotropic samples, including hybrids, have substantially higher effective stiffness than the normal isotropic samples under the same conditions. The graph also indicates that the hybrid samples have higher effective stiffness compared to the normal MRE samples. For example, when there is no magnetic field, the isotropic sample’s effective stiffness is 6.84 kPa, while the anisotropic one has an effective stiffness of 13.59 kPa, which is 98% higher. Further, the stiffness of the hybrid anisotropic sample is 115% higher than the normal anisotropic counterparts. The effective stiffness values for each sample are listed in Table 2.

In addition, the stiffness magnetorheological (SMR) effect was developed to highlight the effect of magnetic flux density on material stiffness. The SMR effect measures the ratio between the effective stiffness modulus caused by the magnet and the material’s original stiffness. In the experiment, the SMR effect is determined by comparing the effective stiffness values when composite materials are statically compressed under the influence of a 160.2 mT magnetic flux density, and to those obtained when the material samples were not exposed to a magnetic field. Thus, the SMR effect can be described as

*SMR* = (*k*max − *kst*)/*kst* × 100% (4)

where *k*max refers to the maximum effective stiffness value measured when the material is exposed to a uniform magnetic flux, and *kst* represents the effective stiffness value when no magnetic flux is applied.

**Figure 8:**
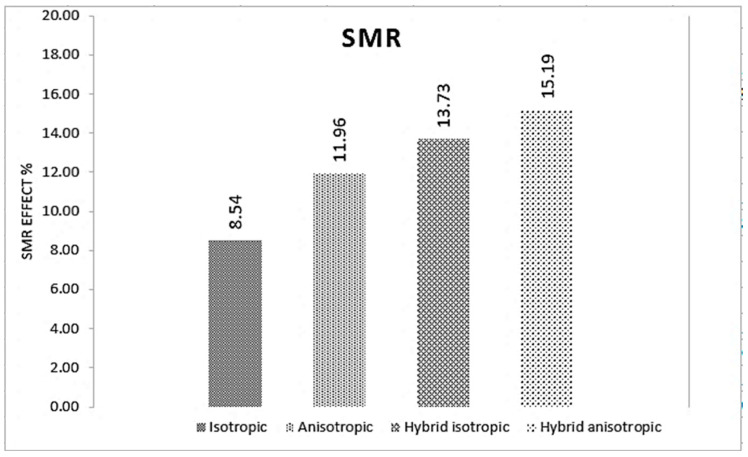
Stiffness magnetorheological (SMR) effect.

When 160.2 mT of magnetic flux density is induced on the material samples, the hybrid anisotropic sample showed the highest proportion of SMR effect improvement, with a value of 15.19%, while the isotropic showed the least, as shown in Figure 8. In general, it can be concluded that the hybrid MRE samples exhibited higher SMR values compared to their normal MRE counterparts.

### 3.4. Dependence of H-MRE-F and MRE Dynamic Properties on the Frequency

The dynamic mechanical properties of the four H-MRE-F and MRE samples were evaluated by measuring the storage modulus and loss modules under variable frequencies from 0.1 to 15.1 Hz at a fixed 2% strain amplitude. The analysis was conducted for all MR samples, with different numbers of magnets (0, 9, and 15 magnets).

**Figure 9:**
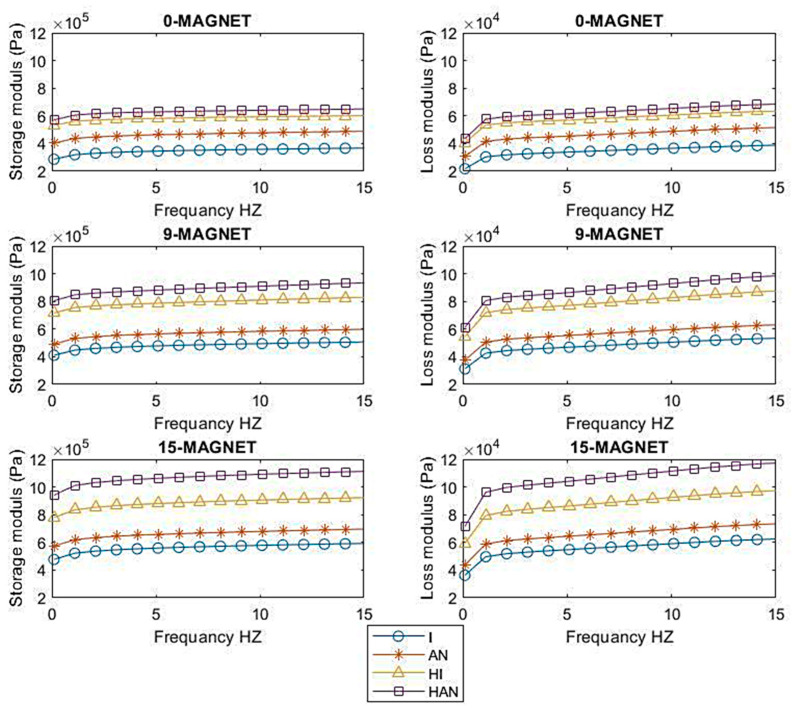
Storage modulus and loss factor of each isotropic, anisotropic MRE, and isotropic and anisotropic H-MREF sample at different magnetic fields.

Figure 9 shows a direct positive correlation between the storage modulus and loss modulus values for all samples. As the frequency varied and an external magnetic field was applied, both the storage and loss modulus gradually increased. Similar observations were reported in previous studies [15]. Furthermore, the frequency of storage and loss modulus rose for all examined samples, indicating that the MRE dynamic characteristics are frequency dependent. It is clear that E’ and E’’ rose roughly linearly with frequency. The same finding was reported in previous studies [15,16]. Furthermore, the increase in $E^{\prime }$ and E’’ is highly influenced by the increase in the number of magnets. The configuration with 15 magnets consistently surpasses both the 9-magnet and 0-magnet configurations in terms of storage modulus across all samples. This trend highlights the consistently superior stiffness exhibited by the 15-magnet configuration, emphasizing its enhanced material performance compared to configurations with fewer magnets. For example, the isotropic storage modules increased from 286 KPa to 367 KPa, and the isotropic storage modulus increased from 403 KPa to 489 KPa. In comparison, the hybrid isotropic storage modulus increased from 533 KPa to 605 KPa and the hybrid anisotropic storage modulus from 571 KPa to 649 KPa, at 0.1 HZ with 0 and 15 magnets, respectively.

**Figure 10:**
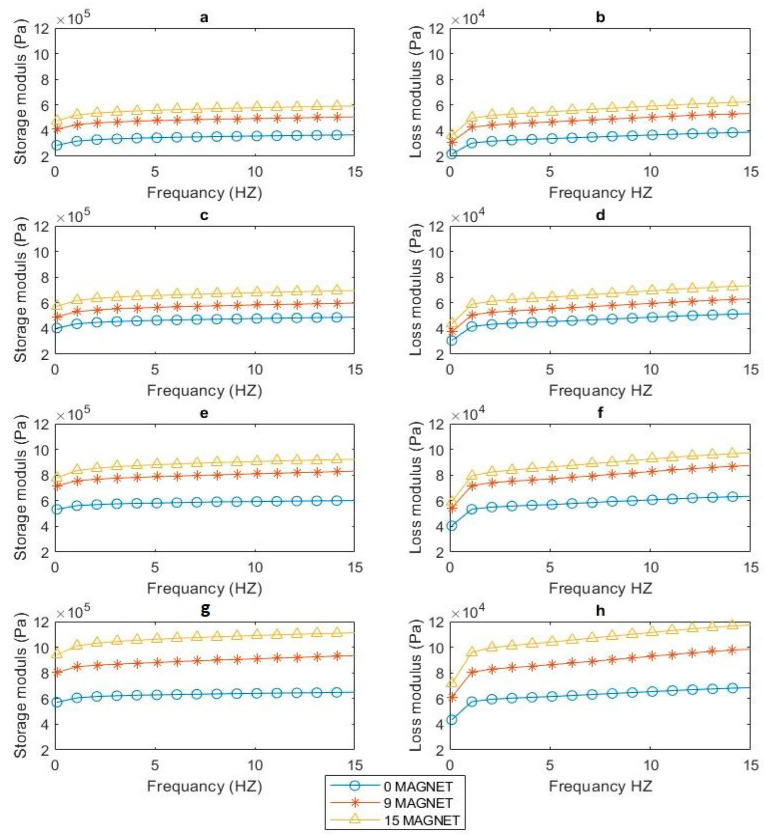
Storage modulus and loss factor for (**a**,**b**) isotropic MRE, (**c**,**d**) anisotropic MRE, (**e**,**f**) hybrid isotropic HMRE-F, and (**g**,**h**) hybrid anisotropic HMRE-F.

Moreover, in Figure 10, the storage modulus (E’) and loss modulus (E’’) of anisotropic samples (Figure 10c,d) surpassed those of isotropic counterparts (Figure 10a,b). Also, by comparing the anisotropic sample, Figure 10c,d, with the hybrid isotropic sample shown in Figure 10e,f, the anisotropic sample shows superiority in both storage modulus (E’) and loss modulus (E’’). This can be attributed to magnetic particle structural alignment during the solidification process in anisotropic materials. This phenomenon, often called the magnetorheological effect, has been demonstrated by many experimental and theoretical investigations [17,18,19,20]. This is due to the generation of antiparticle magnetic attractions when the MRE sample is placed in a magnetic field. Consequently, the magnetic particles migrate from their starting sites in the direction of the external magnetic field, forming a chain-like structure. This chain-like magnetized particle structure acts as a strong reinforcing framework, limiting polymer chain mobility under cyclic loading [21,22,23], thus enhancing the storage modulus of MREs. On the other hand, the hybrid anisotropic illustrated in Figure 10g,h comparatively demonstrated a significant increase in storage modulus (E’) and loss modulus (E’’) values when compared to the other samples. This suggests that the hybrid anisotropic configuration demonstrates a superior response to changes in the magnetic field. The heightened storage modulus and more significant variation in E’ emphasize the enhanced performance and sensitivity of the hybrid anisotropic sample, making it exceptionally responsive to alterations in the magnetic field conditions.

## 4. Conclusions

This paper compares the static and dynamic properties among isotropic, anisotropic, hybrid isotropic, and hybrid anisotropic samples. Observations, dependent on frequency and magnetic field variations, were made using a dynamic mechanical analyzer. The samples underwent compression on a parallel-plate compression apparatus. Static tests measured force and displacement, emphasizing the SMR effect, while frequency sweep measurements examined storage and loss moduli. The force, displacement, storage modulus, and loss modulus were analyzed at various frequencies and magnetic fields.

SEM characterization was employed to examine the microstructure of the manufactured MRE samples. It revealed uniform alignment of CIPs inside the anisotropic samples, while isotropic samples displayed consistent dispersion within the matrix.

The investigation of the static test showed a strong dependence between the force and magnetic field. Further, as the magnetic field increased, all samples exhibited increased force. Anisotropic samples displayed notably higher force levels than their isotropic and hybrid isotropic counterparts at the same displacement. Conversely, the hybrid anisotropic configuration showed the greatest force among all samples for equivalent displacement. The SMR analysis revealed that the hybrid anisotropic sample exhibits the highest percentage, around 117%, while the normal samples showed a relatively lower impact.

The frequency assessments indicated a significant correlation between the moduli and the frequency, where the storage and loss modulus increased with the frequency. The hybrid MRE samples demonstrated a higher storage and loss modulus than the normal ones. This indicates that the hybrid MRE-F, more specifically the hybrid anisotropic sample, is stiffer and absorbs more energy during deformation than the others. In addition to that, the elevation of the magnetic field led to a further increase in the storage and loss moduli, attributed to heightened magnetic interactions.

The findings of this study serve as a guide for selecting the appropriate MRE types by emphasizing the performance of hybrid, normal, isotropic, and anisotropic samples through their respective SMR, storage, and loss modulus. Thus, it provides a basis for material selection in creating MRE-based devices, particularly tunable vibration isolators working in compression modes.

## Figures and Tables

**Table 1:** Properties of the silicone rubber and CIP material.

Material	Properties	
Silicone rubber	Type	Elite Double 32 Fast—Zhermach (Italy)
	Mixing ratio	1:1
	Manual mixing time (min:s)	1:00
	Working time (min:s)	5:00
	Setting time	10:00
	Detail reproduction (μm)	2
	Density (kg/m^3^)	1.06
	Tear resistance (N/mm^2^)	5
	Elastic recovery (%)	99.95%
CIP	Grade	CIP CS
	Hard or soft	Soft
	Particle size (μm)	6.0–7.0
	Coating	-
	Density (g/cm^3^)	~7.89

**Table 2:** The effective stiffness value for all samples with 0, 9, and 15 magnets.

	ISO	AN-ISO	H-ISO	H-AN-ISO
#MAGNETS	EFFECTIVE STIFFNESS (KPA)			
0	7.05	13.59	11.16	14.75
9	7.22	14.10	11.70	15.95
15	7.66	15.22	12.67	16.99

## Data Availability

Data are contained within the article.

## Nomenclatures

MR	Magnetorheological
MREs	Magnetorheological elastomers
CIPs	Carbonyl iron particles
DMA	Dynamic mechanical analyzer
VF	Volume fraction
HISO	Hybrid isotropic
HMRE-F	Hybrid MRE + MRF
MRF	Magnetorheological fluid
ISO	Isotropic
ANISO	anisotropic
SEM	Scanning electron microscope
HANISO	Hybrid anisotropic
